# Study on *Gardeniae Fructus* for treating depression with combination of network pharmacology, metabolomics and gut microbiota

**DOI:** 10.3389/fmicb.2026.1979681

**Published:** 2026-10-28

**Authors:** Yankai Dong, Pin Deng, Dujuan Zhan, Yuan Li, Qing-e Liu

**Affiliations:** 1 Chen Yan College of Traditional Chinese Medicine, Lishui University, Lishui, Zhejiang, China; 2 Institute of Basic Theory for Chinese Medicine, China Academy of Chinese Medical Sciences, Beijing, China; 3 Department of Pharmacology, School of Pharmaceutical Sciences, Cheeloo College of Medicine, Shandong University, Jinan, Shandong, China; 4 College of Agriculture and Biotechnology, Lishui University, Lishui, Zhejiang, China

**Keywords:** depression, *Gardeniae Fructus*, gut microbiota, metabolomics, network pharmacology

## Abstract

**Background:**

Depression is a prevalent and disabling mental disorder with limited therapeutic efficacy and delayed response to conventional antidepressants, highlighting the urgent need for safe and effective alternatives. Gardeniae Fructus (GF), a traditional Chinese medicine with documented neuroprotective properties, has shown rapid antidepressant-like effects, yet its mechanisms remain incompletely understood.

**Methods:**

Depression-like behaviors were induced by reserpine in adult Sprague–Dawley rats. Thirty-five rats were randomly and evenly divided into five groups: control (CON), Model, low-dose GF (GFL, 1.2 g/kg·d), high-dose GF (GFH, 2.4 g/kg·d), and fluoxetine (FLU, 10 mg/kg·d). Depressive behaviors were assessed by sucrose preference test, forced swimming test, open field test, body temperature, and eyelid ptosis tests. Differential metabolites were identified through serum untargeted metabolomics, and metabolic pathways were analyzed using MetaboAnalyst. The anti-depression targets of GF were determined by network pharmacology. A network pharmacology–metabolomics network was constructed in Cytoscape, and interactions between key components and key targets were verified by molecular docking. Gut microbiota of rats in different groups was analyzed by 16S rDNA sequencing, and multi-omics integration analysis was performed.

**Results:**

11 active components of GF and 140 potential targets associated with depression were identified, with quercetin emerging as a key compound. Protein–protein interaction analysis highlighted AKT1, TNF, IL6, VEGFA, and TP53 as core targets. In reserpine-induced depressive rats, GF treatment significantly ameliorated depressive-like behaviors, involving shortened immobility time and enhanced sucrose preference, improved body temperature, and alleviated eyelid ptosis. 16S rRNA sequencing revealed that GF modulated gut microbiota composition, and altering functional pathways including thiamine, butanoate, and fatty acid metabolism. Untargeted metabolomics identified 28 differentially expressed metabolites between GF-treated and model groups, predominantly involving lipids and amino acids. Integrated network and metabolomic analyses pinpointed glycosphingolipid metabolism as a critical pathway, with PTEN as a key target and sphinganine and phytosphingosine as principal metabolites. Molecular docking confirmed stable binding between quercetin and PTEN (binding energy: −7.2 kcal/mol). Correlation analyses demonstrated that Bifidobacterium and Turicibacter exhibited pronounced associations with multiple metabolic alterations.

**Conclusion:**

This integrative approach reveals that GF alleviates depression through multi-target, multi-pathway mechanisms involving regulation of gut microbiota, modulation of glycosphingolipid metabolism, and targeting of PTEN, providing a comprehensive basis for its neuroprotective effects and offering novel insights into Traditional Chinese Medicine-based antidepressant strategies.

## 1 Introduction

Depression, a widespread psychiatric disorder, has been designated by the World Health Organization as the foremost cause of disability (Beurel et al., 2020). As a highly disabling and common chronic disorder, depression affects over 350 million people worldwide (Dong et al., 2021) and is one of the predominant causes of death globally. Depression is characterized by disturbances in neuroimmune, neuroendocrine, neurotransmitter, and metabolic systems (Zhang et al., 2021) and its clinical features consist of sustained sadness, diminished interest, reduced concentration or energy, appetite or sleep disturbances, and profound feelings of worthlessness or guilt (Yap et al., 2021). By 2030, it is anticipated to rank as the foremost contributor to the global burden of disease (Chen et al., 2021; He et al., 2022). Although antidepressants are the first-line treatment for depression, individual responses vary considerably, and 50–60% of patients fail to achieve satisfactory therapeutic effects after antidepressant treatment (Yang et al., 2022). Conventional antidepressants are widely prescribed but their clinical utility is hampered by a delayed onset of action (Ren et al., 2022). Therefore, the development of safe and effective drugs remains an urgent issue in the treatment of depression.

Traditional Chinese Medicine (TCM) denotes a holistic system of diagnosis, pathophysiology, and treatment that employs Chinese herbal medicines and is grounded in knowledge and practical experience accumulated over a long history (Feng et al., 2022), and TCM is of great significance in the prevention and treatment of depression (Xue et al., 2022). *Gardeniae Fructus* (GF), the fruit of *Gardenia jasminoides* Ellis, which is grown in many regions of China, is a medicine-food homology herb (Cao et al., 2021). Previous studies showed that GF exhibited rapid antidepressant-like effects comparable to those of an antidepressant in both acute and chronic mouse models (Zhang et al., 2015) as well as in mice exposed to chronic unpredictable mild stress (Ren et al., 2022).

At present, the strategy of developing single-target therapeutic agents has come under increasing scrutiny, primarily owing to insufficient efficacy and the development of drug resistance. Consequently, natural compounds with selective activity against multiple targets of interest are theoretically predicted to show greater efficacy than single-target agents (Li et al., 2021). As interdisciplinary fields such as bioinformatics and systems biology have flourished, approaches to investigating drug–disease interactions have gradually moved from isolated studies toward systematic and holistic analyses. Accordingly, network-based biomedical investigations have been initiated (Wang et al., 2021). Network pharmacology is an integrative field that combines systems biology theory (Liu et al., 2022), pharmacology, information networks, and computer science, and it utilizes computational modeling and multiple databases to screen drug-related molecular targets and disease-associated targets. By incorporating computational, experimental, and clinical studies, network pharmacology creates favorable opportunities for characterizing TCM and connecting it with cutting-edge modern science and technology (Lai et al., 2020). Nevertheless, network pharmacology suffers from limitations inherent in its dependence on a single computational method and public databases. In isolation, it can merely estimate the probability of compound-target interactions and perform pathway analysis (Zhong et al., 2018). Gut microbiota is regarded as the “second genome” of the human body (Zhang et al., 2022). Prior research has indicated that an intricate relationship exists between the brain and gut microbiota. Both depressed patients and animal models of depression exhibit marked alterations in gut microbiota, implying a close association between gut microbes and depression (Zhang et al., 2021). Increasing evidence indicates that intestinal health regulates emotion and behavior through bidirectional communication along the gut-brain axis. Depression has also been linked to alterations in gut microbial composition (Chen et al., 2019). Furthermore, patients with depression or mice with depressive behaviors are also accompanied by alterations in systemic metabolism (Yang et al., 2026; Jadhav et al., 2026).

In the present work, network pharmacology was integrated with gut microbiota and metabolomics to explore the mechanisms responsible for the antidepressant effects of GF. This combined approach offsets the shortage of experimental verification inherent in network pharmacology and may assist in clarifying the therapeutic principles of TCMs for depression. By determining the key targets and mechanisms through which GF counteracts depression, the present study offers novel perspectives on the neuroprotective properties of GF. Figure 1 presented the workflow of this study.

**Figure 1:**
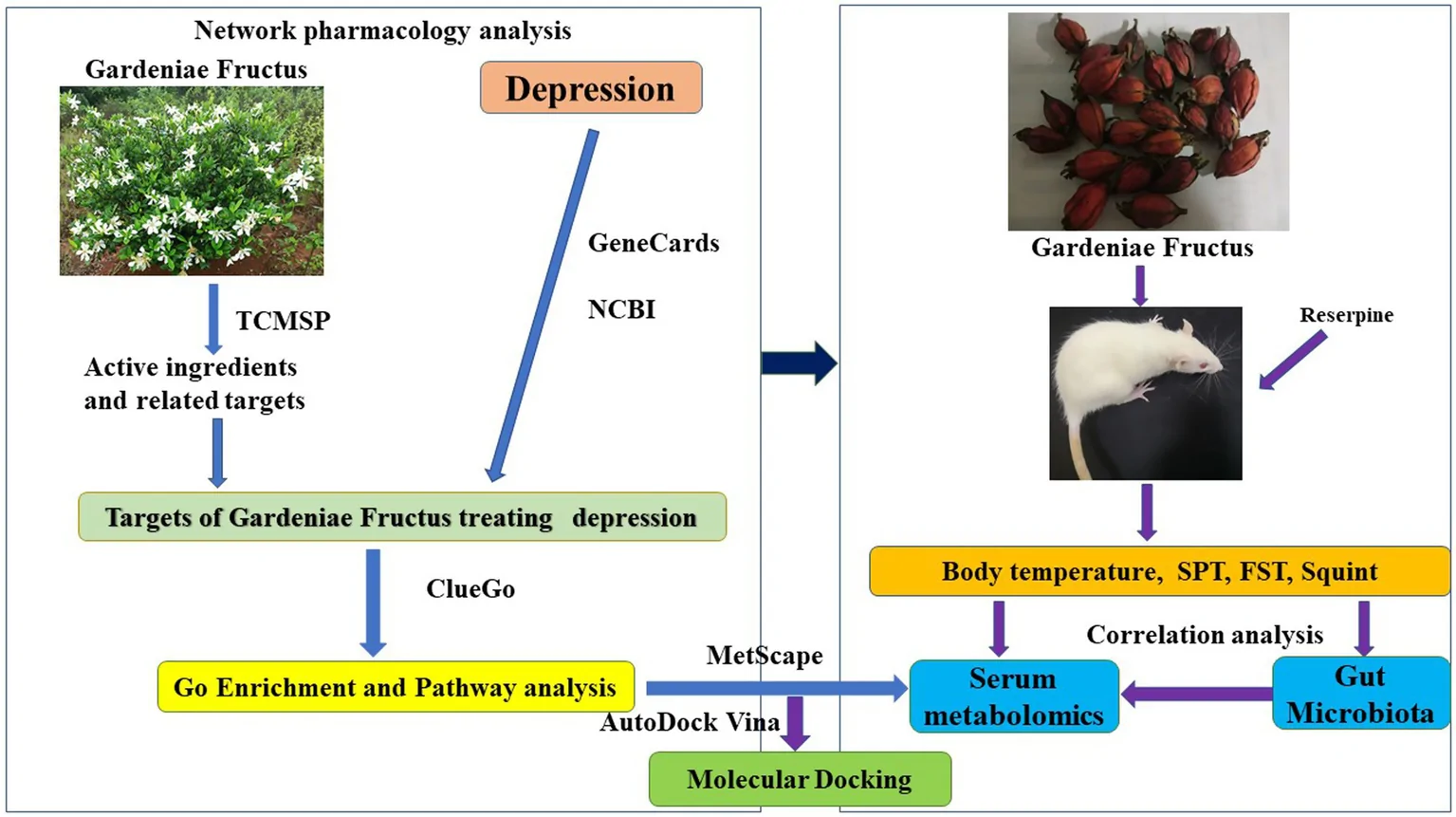
Flowchart of the study.

## 2 Materials and methods

### 2.1 Network pharmacology

#### 2.1.1 Active components and related targets of GF

The Traditional Chinese Medicine Systems Pharmacology Database and Analysis Platform (TCMSP, https://tcmsp-e.com/browse.php?qc=herbs) is specialized systematic pharmacology platform developed for exploring the mechanisms of TCMs. The constituents of GF were obtained from TCMSP. Compounds satisfying the thresholds of oral bioavailability (OB) ≥ 30% and drug-likeness (DL) ≥ 0.18 were regarded as active components, and the targets associated with each component were retrieved (Deng et al., 2021).

#### 2.1.2 Depression-related target genes

Using “depression” and “*Homo sapiens*” as search terms, depression-associated genes were obtained from the National Center for Biotechnology Information (NCBI, https://www.ncbi.nlm.nih.gov/) and GeneCards^1^ (Dong et al., 2025). This search returned 593 genes from NCBI and 3,149 genes from GeneCards (score ≥1) were retrieved. Following the removal of duplicates, 3,223 genes remained.

#### 2.1.3 Candidate targets of GF for depression therapy

A Venn diagram^2^ was used to identify the overlap between GF targets and depression-related targets. The shared targets were considered the target genes through which GF exerts its therapeutic effects on depression.

#### 2.1.4 Development of the active component-target network

Using Cytoscape 3.7.1, the component-target associations were processed and visualized as an “active components-targets” regulatory network.

#### 2.1.5 Protein–protein interaction data

The overlapping genes were uploaded to the STRING database^3^ with *Homo sapiens* selected as the species, and a protein-protein interaction (PPI) network was subsequently constructed. The resulting data were stored in tab-separated values format. The interaction network was then visualized and analyzed with Cytoscape 3.7.1.

#### 2.1.6 Gene ontology and Kyoto encyclopedia of genes and genome pathway enrichment analysis

Gene ontology (GO) and kyoto encyclopedia of genes and genome (KEGG) pathway enrichment analyses of the shared targets were carried out using ClueGO within Cytoscape, with the species set to *Homo sapiens*. Only terms with a *p* < 0.05 were retained for subsequent analysis.

### 2.2 Preparation of GF extract

The GF was purchased from Shanghai Shuofang Pharmaceutical Technology Co. and was identified by the authors themselves as the dried mature fruit of *Gardenia jasminoides* Ellis (Rubiaceae). The resulting powder was subjected to heat reflux extraction with 70% ethanol at a solid-to-liquid ratio of 1:10 (g/mL), each extraction lasting 1.5 h, and this procedure was repeated three times. The pooled extracts were then filtered, concentrated, and made up to volume, and the final solution was kept at 4 °C.

### 2.3 Animals and rearing conditions

Male Sprague–Dawley (SD) rats were procured from SPF (Beijing) Biotechnology Co., Ltd. (Beijing, China). The animals were maintained under standard laboratory conditions at 25 ± 2 °C with a 12-h light/dark cycle and free access to food and water. Before the experiment, all rats were allowed one week to acclimatize. Ethical approval for this study was granted by the Ethics Committee of School of Pharmaceutical Sciences, Shandong University (Approval No. YXDW2025-0159, September 9, 2025).

### 2.4 Experimental grouping, model establishment, and drug administration

After 7-day of acclimation, SD rats were randomly allocated to five groups (n = 7 per group): control (CON), Model, fluoxetine (FLU, 10 mg·kg^−1^ fluoxetine), and low- and high-dose GF groups (GFL and GFH, equivalent to 1.2 and 2.4 g·kg^−1^ crude GF, respectively). The treatment groups were administered their respective doses by intragastric gavage, while equal-volume normal saline was given to the CON and Model groups. Thirty minutes after gavage, all groups except CON received intraperitoneal reserpine (0.2 mg·kg^−1^); the CON group was injected intraperitoneally with an equal volume of normal saline. This regimen was repeated once each day for 14 successive days.

### 2.5 Behavioral assessment in rats

#### 2.5.1 Evaluation of eyelid ptosis

At 1 h after the final reserpine administration, the rats were assessed for the degree of eyelid ptosis. Each rat was placed on a table and scored for eyelid ptosis according to the following criteria: 4, eyelids fully open; 3, one-quarter closure of the eyelids; 2, half-closure of the eyelids; 1, three-quarter closure of the eyelids; 0, complete eyelid closure. The observation time was 15 s per rat.

#### 2.5.2 Measurement of body temperature

Rectal temperature was measured in each rat at 2 h after the final reserpine injection and compared among the groups.

#### 2.5.3 Sucrose preference test

Anhedonia, a core behavioral manifestation of depression, was evaluated by the sucrose preference test (SPT), which was conducted 14 days after drug administration. Twenty-four hours before testing, rats were habituated to a 1% sucrose solution. During habituation, rats housed individually were given two bottles: 100 mL of 1% sucrose solution and an equal volume of water. The positions of the two bottles were exchanged after 12 h of habituation. Before behavioral assessment, rats were subjected to 3 h of water deprivation, followed by a 4-h test. Each rat was then provided with two pre-weighed bottles containing 100 mL of 1% sucrose solution and 100 mL of water, and the bottle positions were reversed after the first 2 h. Sucrose preference was calculated as (sucrose intake / total intake) × 100%.

#### 2.5.4 Forced swimming test

The forced swimming test (FST) was used to evaluate despair-like behavior. Testing was carried out in an open cylinder (height: 60 cm; diameter: 20 cm) filled with water to a depth of 40 cm, and the water temperature was kept at 23–25 °C throughout. Each rat underwent a 6-min trial in the cylinder individually, during which the immobility duration was recorded.

#### 2.5.5 Open field test

The open field test (OFT) was carried out in a square arena. Each rat was gently positioned in the center of the arena, after which the following parameters were documented: the number of center crossings (only when all four paws crossed a center), rearing frequency (the number of times the animal stood on its hind legs), and grooming behavior (cleaning of the body surface to remove oil, debris, and dirt). Following each trial, the rat was placed back into its home cage, and the arena was wiped thoroughly with 75% ethanol to eliminate residual odors and prevent interference with subsequent animals.

### 2.6 Characterization of gut microbiota

#### 2.6.1 Fecal sampling and DNA extraction

24 h after the last gavage, fresh feces samples were collected from rats into sterile Eppendorf tubes, flash-frozen in liquid nitrogen, and kept at −80 °C. Extraction of total DNA was carried out with the E.Z.N.A.^®^ Soil DNA Kit (Omega Bio-tek, GA, United States), followed by determination of DNA concentration and purity.

#### 2.6.2 16S rDNA gene sequencing

PCR amplification of the V3-V4 region of 16S rDNA was carried out using the forward primer (ACTCCTACGGGAGGCAGCAG) and reverse primer (GGACTACHVGGGTWTCTAAT). The amplification mixture was constituted as below: 5 × FastPfu Buffer, 4 μL; 2.5 mM dNTPs, 2 μL; forward primer (5 μM), 0.8 μL; reverse primer (5 μM), 0.8 μL; FastPfu Polymerase, 0.4 μL; BSA, 0.2 μL; template DNA, 10 ng; ddH₂O was supplemented to reach a final volume of 20 μL. PCR cycling was conducted as follows: preliminary denaturation at 95 °C for 3 min; then 30 cycles of 30 s at 95 °C, 30 s at the annealing temperature, and 45 s at 72 °C; and terminal extension at 72 °C for 10 min, after which samples were maintained at 10 °C until manually terminated. The resultant PCR products were evaluated by 2% agarose gel electrophoresis. PCR products were examined and quantified with a Quantus™ Fluorometer. In accordance with the sequencing quantity requirements for each sample, the products were combined in appropriate ratios. MiSeq library preparation was conducted with the NEXTFLEX Rapid DNA-Seq Kit, and sequencing was performed on Illumina’s MiSeq PE300/NovaSeq PE250 platform, fastp (https://github.com/OpenGene/fastp, version 0.20.0) was employed for quality filtering of the raw sequencing reads, and FLASH (http://www.cbcb.umd.edu/software/flash, version 1.2.7) was applied for read merging. UPARSE (http://drive5.com/uparse/, version 7.1) was used to group sequences into OTUs at 97% similarity. Rarefaction curves serve to determine whether the sequencing depth of each sample and the sample size are adequate to reflect the microbial diversity in community samples; a flatter curve indicates more adequate sequencing. Alpha diversity analysis, comprising determination of the Chao, ACE, Shannon, and Simpson indices, was applied to assess abundance and diversity. Beta diversity analysis displayed the natural distribution of samples, indicating the similarity among samples. LEfSe analysis was employed to identify biomarkers according to linear discriminant analysis (LDA) effect size (LDA > 2).

### 2.7 Metabolomic analysis

#### 2.7.1 Gathering of serum samples

Following the behavioral experiments, rats in each group were anesthetized with 1% sodium pentobarbital via intraperitoneal injection (40 mg/kg, 0.4 mL/100 g). Blood samples were acquired by ocular enucleation and centrifuged at 4,000 rpm at 4 °C for 15 min. The supernatant was harvested, flash-frozen in liquid nitrogen, and maintained at −80 °C pending subsequent analysis.

#### 2.7.2 Metabolite extraction

Serum samples were taken out from −80 °C storage and thawed at 4 °C. For each sample, 40 μL of serum was moved to a new centrifuge tube, after which pre-cooled mixed organic solvent (acetonitrile:methanol = 1:1, volume) was added. After homogenized by vortexing for 1 min, mixture was centrifuged at 4 °C and 12,000 rpm for 10 min. Supernatant was moved into a new centrifuge tube. The solvent was dried using a freeze dryer, and remnant was reconstituted with 100 μL of 50% acetonitrile. After filtration through a 0.22 µm organic filter membrane, the extraction was transferred into an autosampler vial. An aliquot of 5 μL from each sample was pooled to prepare a quality control sample, which was then transferred into an autosampler vial for UHPLC–MS/MS analysis.

#### 2.7.3 UHPLC–MS/MS analysis

Chromatographic separation was achieved on an ACQUITY UPLC BEH C18 column (100 mm × 2.1 mm, 1.7 μm, Waters, United States). The column was maintained at 35 °C with a flow rate of 0.2 mL/min. The mobile phases were composed of (A) 0.1% formic acid in ultrapure water and (B) 0.1% formic acid in acetonitrile. Elution was carried out with the following gradient: 0–2 min, 5% B; 2–6 min, 5–20% B; 6–12 min, 20–50% B; 12–16 min, 50–80% B; 16–17 min, 80–95% B; 17–20 min, 95% B; 21–25 min, 5% B. The injection volume was 2 μL, and data were collected in both positive and negative ion modes.

Mass spectrometric acquisition was performed in full MS/dd-MS^2^ mode. The MS^1^ scan range was set to 80–1,200 m/z, and the eight most intense ions were chosen for fragmentation and subsequent MS^2^ scanning. The desolvation gas and sheath gas flows were 35 Arb and 10 Arb, respectively. The ion transfer tube and desolvation gas temperatures were maintained at 325 °C and 350 °C, respectively. Spray voltages of 3,800 V (positive mode) and 3,500 V (negative mode) were applied. For MS^1^, the resolution was 70,000 FWHM, the AGC target was 1e6, and the maximum injection time was 50 ms. For MS^2^, the resolution was 17,500 FWHM, the AGC target was 1e5, and the maximum injection time was 50 ms. Fragmentation was conducted using higher-energy C-trap dissociation with normalized collision energies of 20, 40, and 60 eV.

#### 2.7.4 Data preprocessing, database searching and analysis

The raw UHPLC–MS/MS dataset was processed with Progenesis QI (version 2.2), after which metabolite identification was performed. A polynomial regression signal correction method was then applied for data correction. The corrected data matrix was loaded into SIMCA-P for principal component analysis (PCA), orthogonal partial least squares discriminant analysis (OPLS-DA), and permutation testing. Variable importance in projection (VIP) values were derived from the OPLS-DA model, subsequently, a t-test was performed to determine statistical significance, and metabolites meeting the criteria of *p* < 0.05 and VIP > 1 were regarded as differential metabolites. These differential metabolites were annotated by searching the Human Metabolome Database^4^, LIPID MAPS^5^, METLIN^6^, and the KEGG database.^7^ Finally, the metabolic pathways linked to these differential metabolites were analyzed via the MetaboAnalyst online platform.

Serum differential metabolites obtained from metabolomic profiling and network pharmacology-predicted targets of GF against depression were merged in Cytoscape 3.7.1 with the MetScape plugin, generating a comprehensive compound-reaction-enzyme-gene network. By combining these molecular targets with the differential metabolites, core regulatory targets and key metabolic biomarkers were identified. The relationship between group-differential metabolites and key gut microbiota was assessed using the Hmisc package in R.

#### 2.7.5 Molecular docking

Target protein crystal structures were downloaded from the RCSB Protein Data Bank^8^, whereas ligand files were retrieved from the TCMSP database. Using AutoDockTools 1.5.6, both ligand and core target structures were transformed into PDBQT format. Prior to docking, proteins were prepared by deleting water molecules and introducing polar hydrogens. Docking simulations were conducted in AutoDock Vina, employing a genetic algorithm to compute binding affinities. The resulting optimal conformations were ultimately visualized with Discovery Studio 2019.

### 2.8 Statistical analysis

One-way ANOVA was employed for statistical evaluation. Values were reported as the mean ± standard deviation. A difference among groups was regarded as statistically significant at *p* < 0.05.

## 3 Results

### 3.1 Network pharmacology analysis

#### 3.1.1 Main active ingredients of GF

The TCMSP database provided 98 components of GF in total. Among them, 15 met the screening thresholds of OB ≥ 30% and DL ≥ 0.18 (Figure 2A). After excluding components without corresponding targets, 11 compounds were retained and were listed in Table 1.

**Figure 2:**
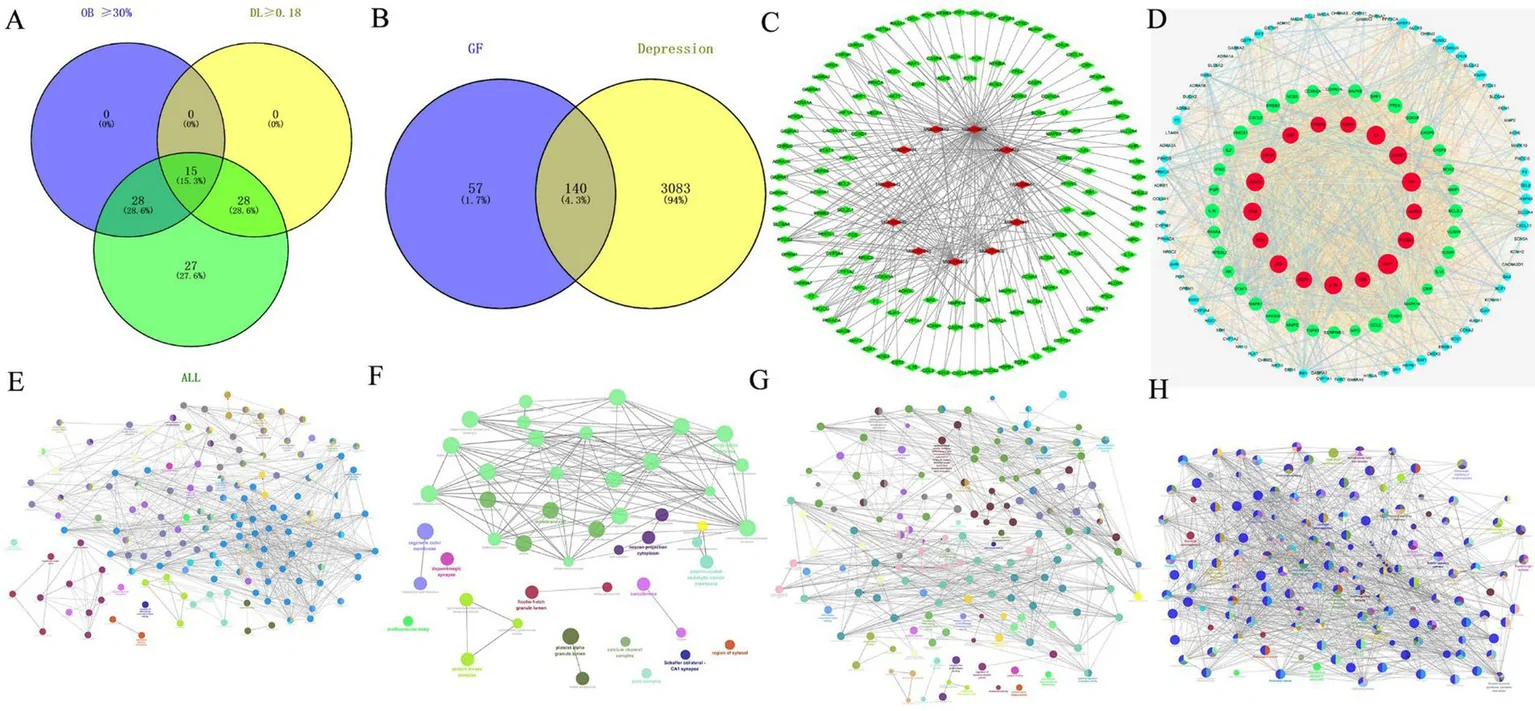
Network pharmacology analysis of the antidepressant effects of GF. **(A)** Venn diagram of GF compounds. **(B)** Venn diagram of GF- and depression-related targets. **(C)** Active component-target network. Red nodes represent active components, and green nodes represent targets. **(D)** Protein–protein interaction network. Enriched GO terms for biological process **(E)**, cellular component **(F)**, and molecular function **(G)** analyses. **(H)** KEGG pathway enrichment analysis.

**Table 1:** Active ingredients of GF.

Rank	Mol ID	Molecule name	MW	OB (%)	DL
1	MOL001406	crocetin	328.44	35.3	0.26
2	MOL001941	Ammidin	270.3	34.55	0.22
3	MOL004561	Sudan III	352.42	84.07	0.59
4	MOL000098	quercetin	302.25	46.43	0.28
5	MOL000358	beta-sitosterol	414.79	36.91	0.75
6	MOL000422	kaempferol	286.25	41.88	0.24
7	MOL000449	Stigmasterol	412.77	43.83	0.76
8	MOL001494	Mandenol	308.56	42	0.19
9	MOL001942	isoimperatorin	270.3	45.46	0.23
10	MOL003095	5-hydroxy-7-methoxy-2-(3,4,5-trimethoxyphenyl) chromone	358.37	51.96	0.41
11	MOL007245	3-Methylkempferol	300.28	60.16	0.26

#### 3.1.2 Targets of GF for treating depression

In total, 197 targets of GF were acquired based on TCMSP database, and 3,223 depression-related targets were obtained according to NCBI and GeneCards. Ultimately, 140 targets of GF involved in the treatment of depression were identified, as shown in Figure 2B.

#### 3.1.3 Active component-target network analysis

An “active component-target” network was generated with Cytoscape. In this work, 11 components were retrieved via the TCMSP database. Active components were represented as red nodes and potential targets as green nodes. The edges indicated the associations between active ingredients and their corresponding targets, thereby indicating the multicomponent and multitarget properties of GF (Figure 2C).

#### 3.1.4 PPI network analysis

With STRING tool, a preliminary PPI network was constructed with 140 commmon genes. As shown in Figure 2D, the network consisted of 2,459 edges and 139 nodes, with an average node degree of 35.4 and a local clustering coefficient of 0.619. Node size represented target degree. Proteins with high degree values played crucial roles within the network, and the five top-ranked proteins by degree were AKT1, TNF, IL6, VEGFA, and TP53.

#### 3.1.5 GO and KEGG pathway enrichment analysis

GO enrichment analysis yielded a total of 1,650 GO terms, encompassing 1,250 biological process terms (Figure 2E), 47 cellular component terms (Figure 2F), and 208 molecular function terms (Figure 2G). The research findings indicated that main GO terms associated with depression included transmitter-gated ion channel activity involved in regulation of postsynaptic membrane potential, dopaminergic synapse, dopamine catabolic process, synaptic signaling, regulation of synaptic transmission, glutamatergic. These terms are mechanistically linked to depression. “Dopaminergic synapse” and “dopamine catabolic process” indicate disturbances in dopamine homeostasis within reward and motivational circuits, which are closely associated with anhedonia in depression. “Synaptic signaling” and “regulation of synaptic transmission, glutamatergic” reflect alterations in excitatory synaptic transmission and synaptic plasticity, which are increasingly recognized as core features of stress-related depressive pathophysiology. These GO terms highlight neurotransmitter imbalance and synaptic dysfunction as key mechanisms of depression.

KEGG pathway enrichment analysis produced 145 pathways in total. Figure 2H presented the key items screened based on *p*-value. Enriched signaling pathways primarily included IL-17 signaling pathway (*p* = 1.0016E-18, amount of molecules: 21), TNF signaling pathway (*p* = 2.74E-18, amount of molecules: 22), MAPK signaling pathway (*p* = 2.3701E-16, amount of molecules: 30), HIF-1 signaling pathway (*p* = 9.8517E-14, amount of molecules: 18), and PI3K-Akt signaling pathway (*p* = 2.6172E-13, amount of molecules: 29).

### 3.2 Antidepressant effects of GF on behavior

The behavioral results were presented in Figure 3. In the FST, immobility duration was significantly prolonged in Model group compared with CON group (*p* < 0.05), while treatment with GFL, GFH, or FLU significantly shortened immobility time relative to Model group (*p* < 0.05) (Figure 3A). In SPT, the sucrose preference percentage was significantly reduced in Model group in comparison with CON group (*p* < 0.05) and was significantly increased in all treatment groups compared with Model group, particularly in GFH group (*p* < 0.05) (Figure 3B). In the OFT, the Model group exhibited a significantly decreased rearing frequency (Figure 3C) and altered grooming frequency (Figure 3D). Rearing frequency in the GFH group and grooming frequency in the FLU group were significantly increased, whereas no significant variation with respect to the number of central zone crossings was detected among groups (Figure 3E). In addition, Model group exhibited a significant reduction in body temperature (Figure 3F) and a reduced eyelid ptosis score (Figure 3G) compared with the CON group (*p* < 0.05). Administration of GFL, GFH, or FLU significantly rose body temperature and eyelid ptosis score in contrast to Model group (*p* < 0.05). Taken together, these findings indicated that GF ameliorated depression-like behavioral triggered by reserpine in rats. Behavioral tests demonstrated that GFH exerted the most pronounced antidepressant-like effects; therefore, samples from the CON, Model, and GFH groups were included in the subsequent gut microbiota and metabolomics analyses.

**Figure 3:**
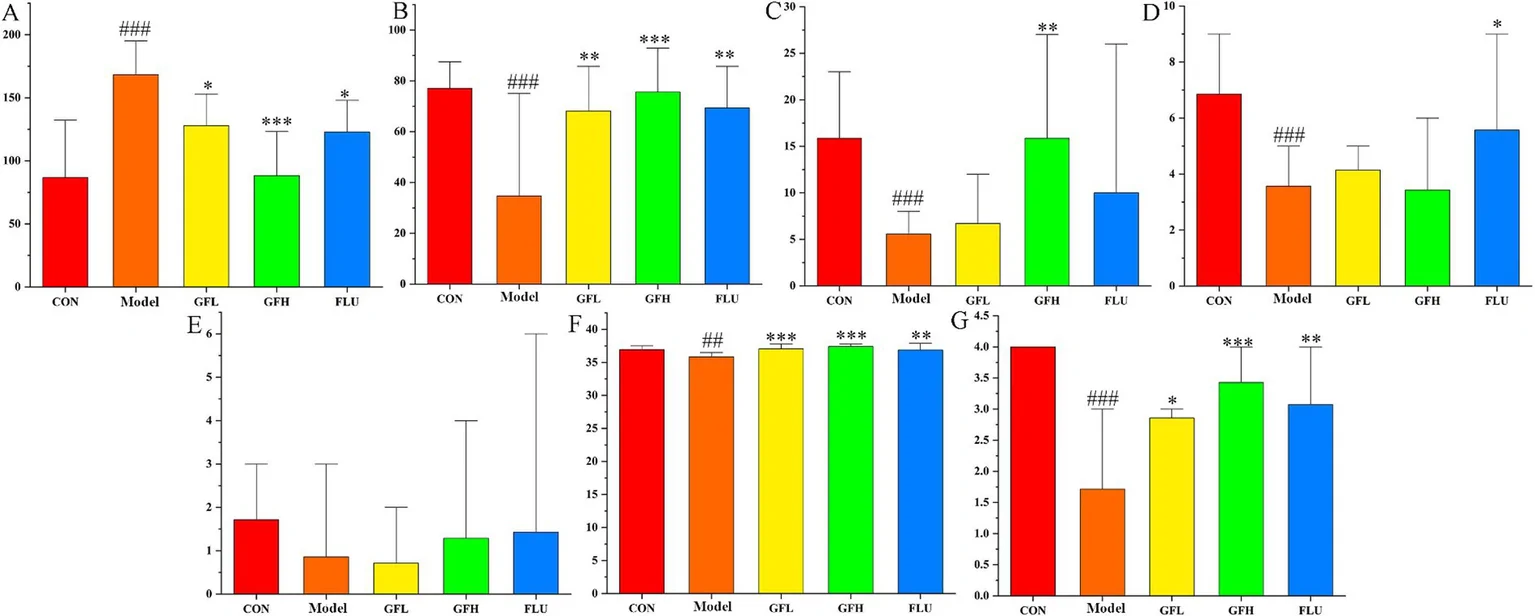
Behavioral tests evaluating the amelioration of depressive-like behaviors by GF. **(A)** Forced swimming test. **(B)** Sucrose preference test. Open field test: **(C)** Rearing frequency. **(D)** Grooming frequency. **(E)** Number of central zone crossings. **(F)** Body temperature monitoring. **(G)** Eyelid ptosis.

### 3.3 Profiling of gut microbiota

#### 3.3.1 Characteristics of gut microbiota

An OTU was defined as an artificially delineated taxon for phylogenetic or population genetics analyses. In general, sequences sharing more than 97% similarity were clustered grouped into a common OTU, with each OTU represented by a representative sequence (Meng et al., 2020). Alpha diversity was used to characterize species abundance and diversity, and Chao, ACE, Shannon, Simpson, Sobs, and Coverage indices were commonly used metrics. The Sobs, Chao, and ACE indices reflected species abundance, whereas Shannon and Simpson indices indicated species variety. The Coverage index represented the sequencing coverage of each sample. Figure 4A showed that the rarefaction curves of all samples plateaued once the sequencing depth reached saturation. This indicated that the current sequencing depth was sufficient, as it effectively captured the majority of bacterial diversity and further sequencing would yield only a minimal number of additional OTUs. Chao index was lower in GFH group than in Model group, suggesting slightly lower species richness in the GFH group. In addition, the remaining indices exhibited no significant differences among the groups, indicating comparable alpha diversity (Figure 4B).

**Figure 4:**
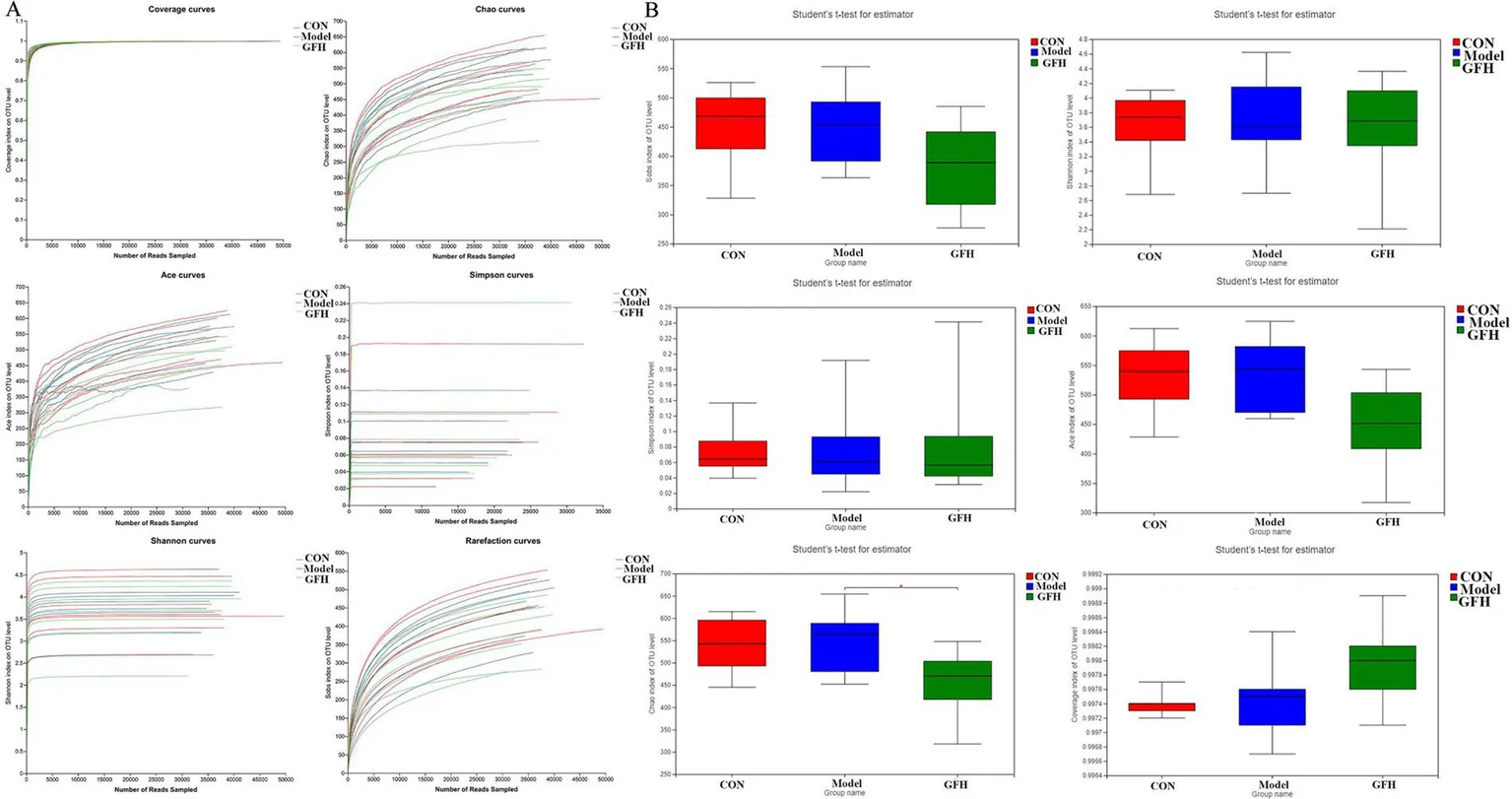
Alpha diversity analysis. **(A)** Rarefaction curves based on multiple diversity indices. **(B)** Comparison of alpha diversity among groups.

#### 3.3.2 Analysis of community composition

To gain further insight into the global structural changes in gut microbiota across different groups, principal coordinate analysis and non-metric multidimensional scaling were conducted. As shown in Figures 5A,B, samples were clearly separated among the different. Figure 5C showed that intergroup differences were greater than intragroup differences. The predominant species across various taxonomic ranks were presented in Figures 5D–H. In terms of phylum level, the predominant taxa were Firmicutes, Bacteroidota, Actinobacteriota, Patescibacteria, Desulfobacterota, Proteobacteria, and Verrucomicrobiota (Figure 5D). At the class level, the dominant species were Bacilli, Clostridia, Bacteroidia, Actinobacteria, Coriobacteria, Saccharimonadia, Negativicutes, Desulfovibrionia, Gammaproteobacteria, and Verrucomicrobiae (Figure 5E). At the order level, the dominant species included Lactobacillales, Bacteroidales, Lachnospirales, Oscillospirales, Peptostreptococcales-Tissierellales, Erysipelotrichales, Bifidobacteriales, Coriobacteriales, among others (Figure 5F). In terms of family, the predominant taxa were Lactobacillaceae, Muribaculaceae, Lachnospiraceae, Peptostreptococcaceae, Erysipelotrichaceae, Oscillospiraceae, Bifidobacteriaceae, Eggerthellaceae, and norank_o_Clostridia_UCG-014, among others (Figure 5G). In terms of genus, the dominant species comprised Lactobacillus, norank_f_Muribaculaceae, Romboutsia, unclassified_f_Lachnospiraceae, Lachnospiraceae_NK4A136_group, Bifidobacterium, Allobaculum, norank_f_norank_o_Clostridia_UCG-014, UCG-005, Clostridium_sensu_stricto_1, Dubosiella, Streptococcus, Candidatus_Saccharimonas, and Monoglobus, among others (Figure 5H).

**Figure 5:**
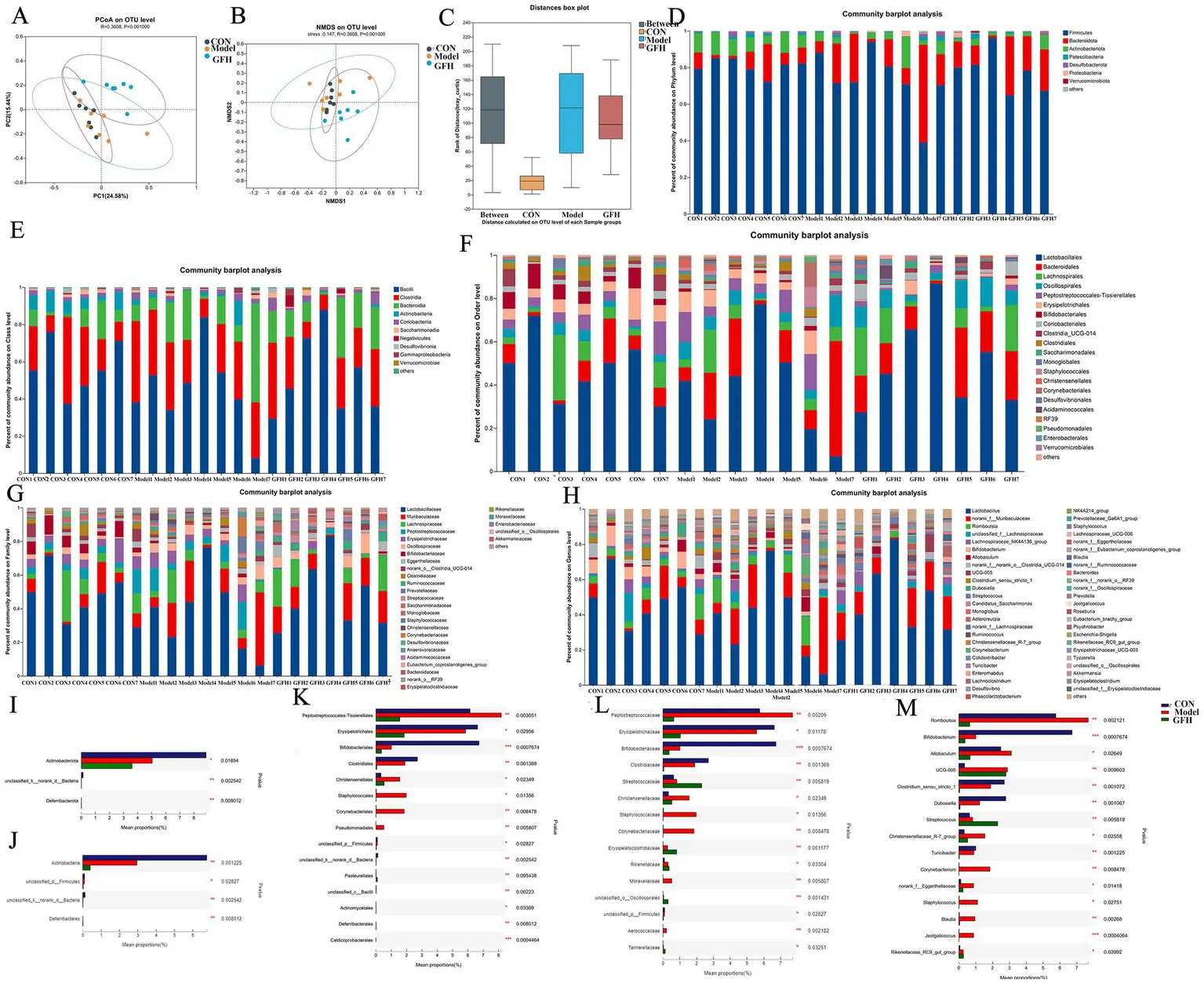
Beta diversity and taxonomic composition analysis. **(A)** Principal coordinates analysis score plot. **(B)** Non-metric multidimensional scaling score plot. **(C)** ANOSIM analysis. Dominant taxa at different taxonomic levels: **(D)** phylum, **(E)** class, **(F)** order, **(G)** family, and **(H)** genus. Differentially abundant taxa at different taxonomic levels: **(I)** phylum, **(J)** class, **(K)** order (top 15), **(L)** family (top 15), and **(M)** genus (top 15).

The differential species at different taxonomic levels were shown in Figures 5I–M. At the phylum level, the differential species were Actinobacteriota, unclassified_k_norank_d_Bacteria, and Deferribacterota (Figure 5I). At the class level, the differential species were Actinobacteria, unclassified_p_Firmicutes, unclassified_k_norank_d_Bacteria, and Deferribacteres (Figure 5J). At the order level, the differential species included Peptostreptococcales-Tissierellales, Erysipelotrichales, Bifidobacteriales, Clostridiales, Christensenellales, Staphylococcales, Corynebacteriales, Pseudomonadales, among others (Figure 5K). At the family level, the differential species included Peptostreptococcaceae, Erysipelotrichaceae, Bifidobacteriaceae, Clostridiaceae, Streptococcaceae, Christensenellaceae, Staphylococcaceae, Corynebacteriaceae, Erysipelatoclostridiaceae, and Rikenellaceae, among others (Figure 5L). At the genus level, the differential species included Romboutsia, Bifidobacterium, Allobaculum, UCG-005, Clostridium_sensu_stricto_1, Dubosiella, Streptococcus, Christensenellaceae_R-7_group, and Turicibacter, among others (Figure 5M).

To explore the crucial gut microbiota in each group, LEfSe analysis of taxon abundance was performed. As shown in Figures 6A,B, 50, 74, and 45 key species were identified in the CON, Model, and GFH groups, respectively.

**Figure 6:**
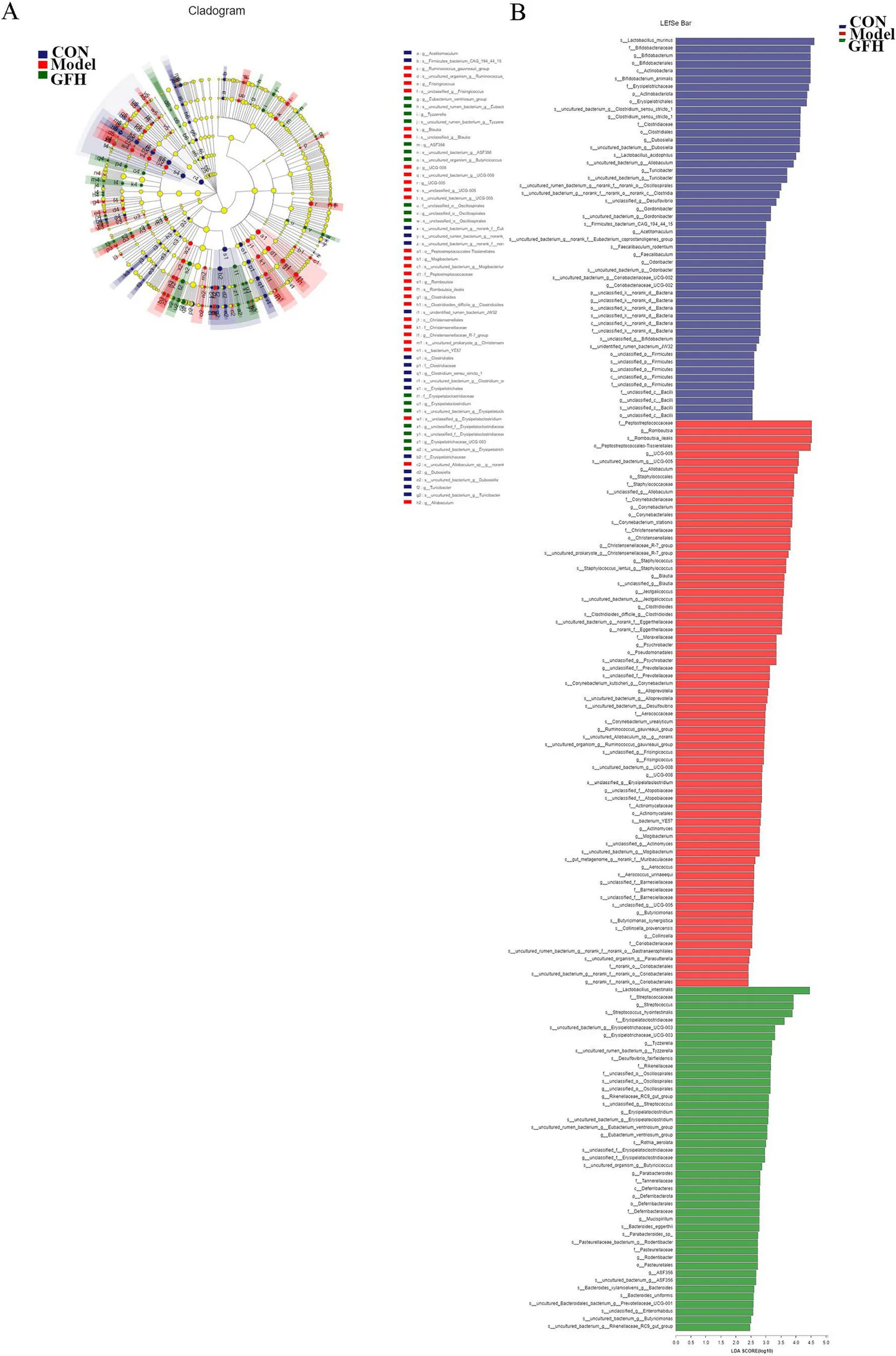
LEfSe analysis. **(A)** Cladogram illustrating group-wise differences in the gut microbiota. **(B)** Histogram of LDA scores.

#### 3.3.3 Functional prediction of gut microbiota

The functions of the gut microbiota were predicted, and the pathways that exhibited significant differences among groups were presented in Figure 7. In the GFH group, the significantly upregulated pathways included Thiamine metabolism, Butanoate metabolism, Nicotinate and nicotinamide metabolism, Oxidative phosphorylation, Carbon metabolism, Metabolic pathways, Cysteine and methionine metabolism, Fatty acid metabolism, Fatty acid biosynthesis, Prodigiosin biosynthesis, Biotin metabolism, Lysine degradation, Styrene degradation, Pathways in cancer, beta-Alanine metabolism, Phosphonate and phosphinate metabolism, and cAMP signaling pathway, among others. The significantly downregulated pathways in the GFH group included: MAPK signaling pathway—yeast, Carotenoid biosynthesis, Lipoarabinomannan (LAM) biosynthesis, Biosynthesis of unsaturated fatty acids, Toluene degradation, Thyroid hormone signaling pathway, Human papillomavirus infection, Viral carcinogenesis, Type II diabetes mellitus, Starch and sucrose metabolism, Proteasome, Chagas disease (American trypanosomiasis), Carbohydrate digestion and absorption, and Pancreatic secretion.

**Figure 7:**
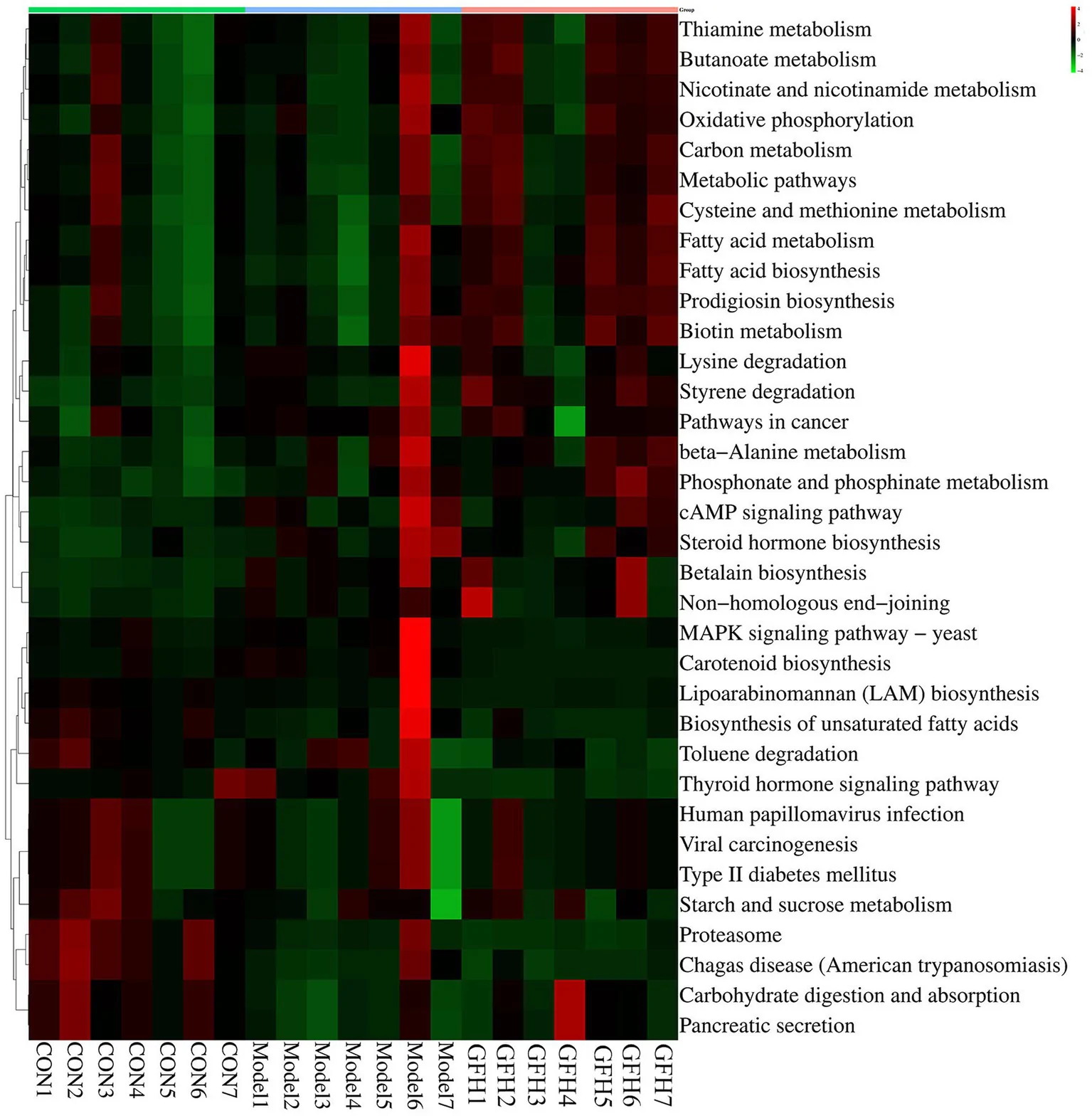
Functional analysis of the gut microbiota.

### 3.4 Metabolomics analysis

#### 3.4.1 Untargeted metabolomic profiling

A total of 4,814 serum metabolites were identified. To assess global metabolic alterations, PCA and OPLS-DA were performed on the CON, Model, and GFH groups. In positive ion mode, both PCA (Figure 8A) and OPLS-DA (Figure 8B) score plots exhibited well-defined clustering, with pronounced intergroup separation and strong intragroup cohesion. The robustness of the OPLS-DA models was further confirmed by permutation tests (Figure 8C), which demonstrated stability and excluded overfitting. Similarly, pairwise comparisons between Model and CON (Figures 8D–F) and between GFH and Model (Figures 8G–I) revealed clear discriminative trends in PCA and OPLS-DA score plots, with permutation tests consistently indicating stable, non-overfitted models. Comparable metabolic discrimination was also observed in negative ion mode (Figure 9), in which PCA and OPLS-DA score plots revealed significant shifts in metabolic signatures across the three groups, supported by consistent permutation test results.

**Figure 8:**
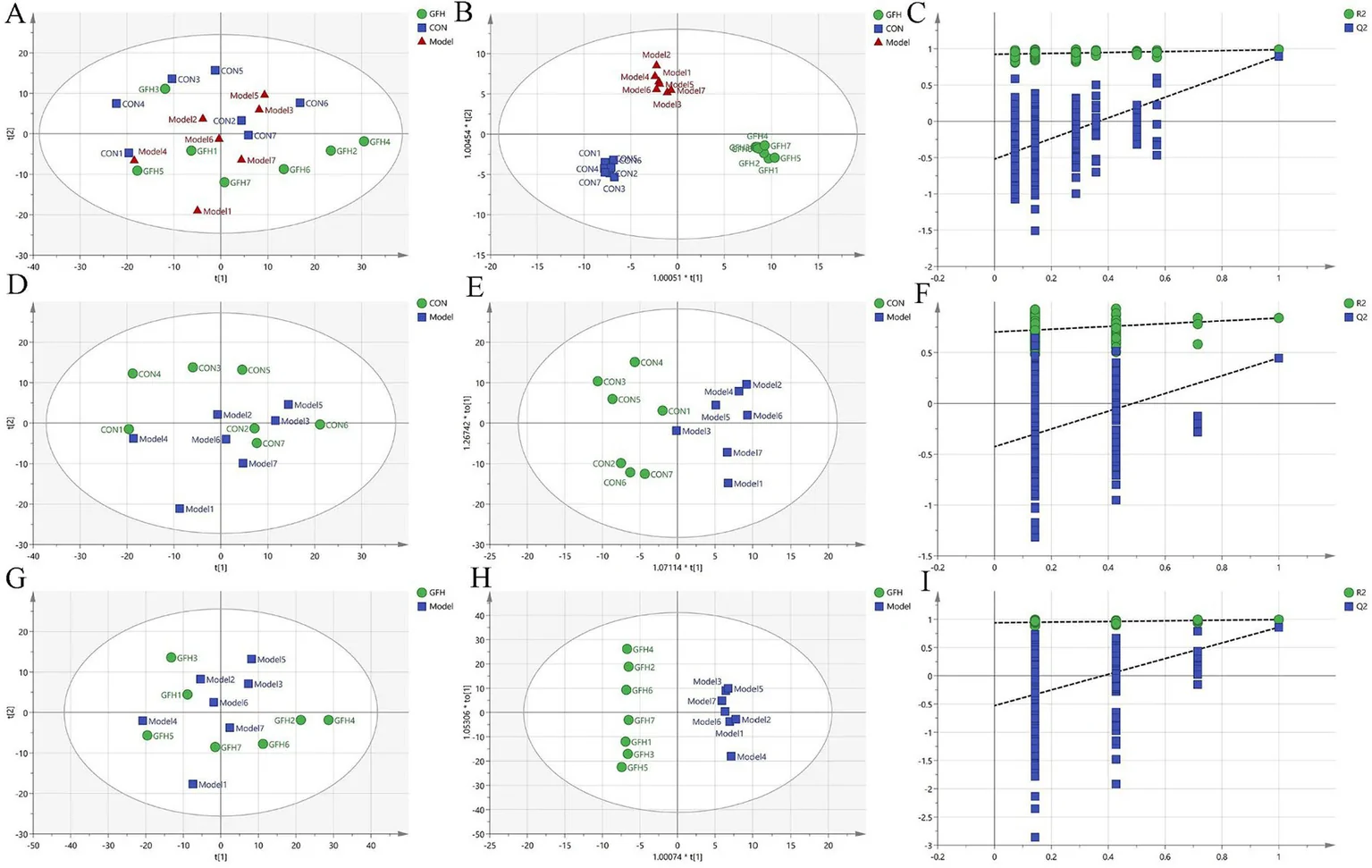
PCA and OPLS-DA score plots of serum metabolites in positive ion mode. Three groups of **(A)** PCA score plot. **(B)** OPLS-DA score plot. **(C)** Permutation test for the OPLS-DA model. Model vs. CON of **(D)** PCA score plot. **(E)** OPLS-DA score plot. **(F)** Permutation test for the OPLS-DA model. GFH vs. Model of **(G)** PCA score plot. **(H)** OPLS-DA score plot. **(I)** Permutation test for the OPLS-DA model.

**Figure 9:**
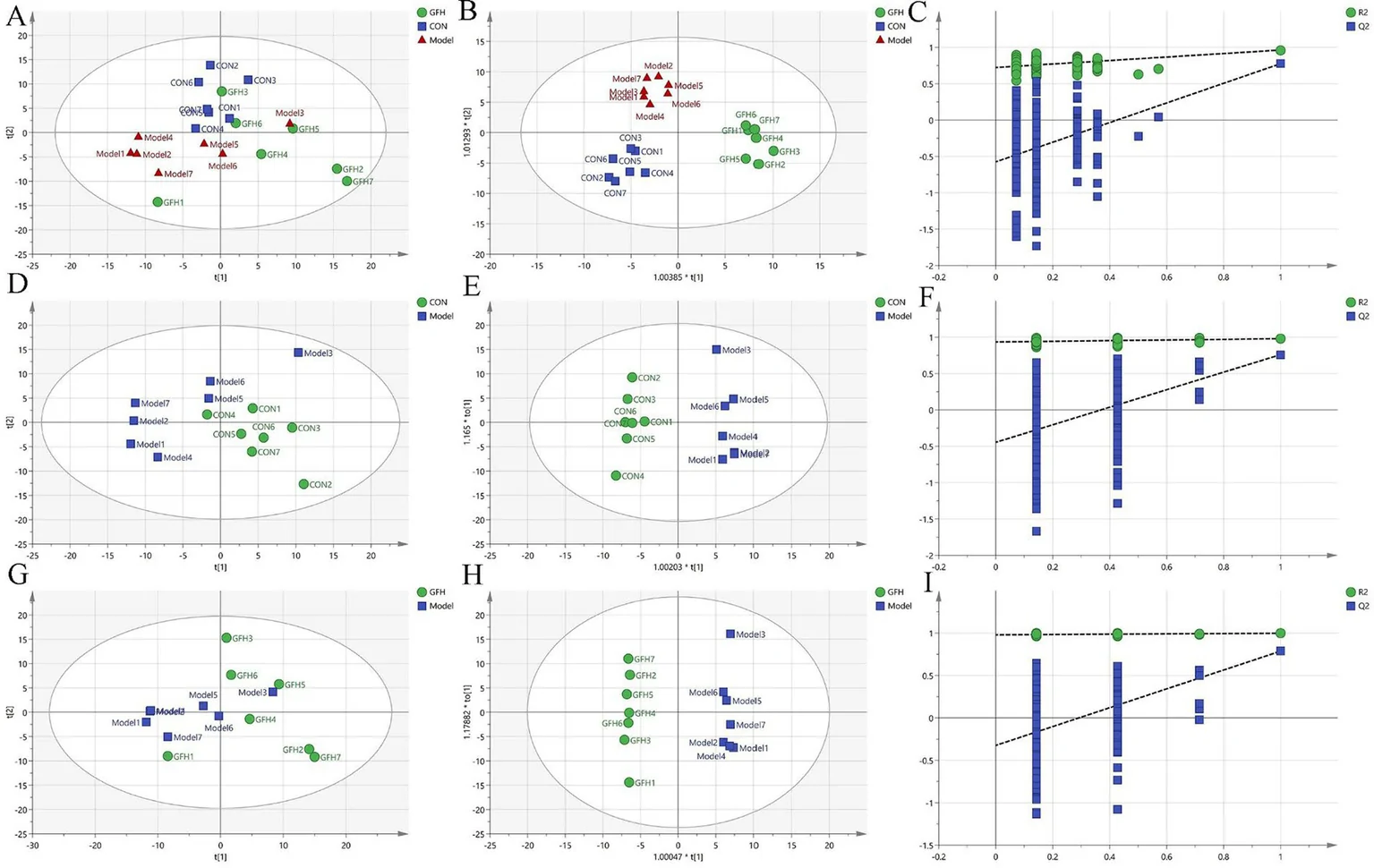
PCA and OPLS-DA score plots of serum metabolites in negative ion mode. Three groups of **(A)** PCA score plot. **(B)** OPLS-DA score plot. **(C)** Permutation test for the OPLS-DA model. Model vs. CON of **(D)** PCA score plot. **(E)** OPLS-DA score plot. **(F)** Permutation test for the OPLS-DA model. GFH vs. Model of **(G)** PCA score plot. **(H)** OPLS-DA score plot. **(I)** Permutation test for the OPLS-DA model.

#### 3.4.2 Differential metabolites and pathway analysis

Using the criteria of VIP > 1 and *p* < 0.05, we identified 29 differential metabolites in serum between the Model and CON groups (Figure 10A; Table 2). A strong positive correlation was observed among most of these metabolites (Figure 10B), and enrichment analysis identified “Metabolism of xenobiotics by cytochrome P450” as the predominant pathway involved (Figure 10C). For the GFH versus Model comparison, 28 differential metabolites were detected (Figure 10D; Table 3), and their correlation matrix is shown in Figure 10E, with most metabolite-metabolite relationships being positive. Subsequent pathway analysis further revealed that the most significantly enriched pathways included terpenoid backbone biosynthesis, arginine and proline metabolism, glycerophospholipid metabolism, and pentose and glucuronate interconversions (Figure 10F).

**Figure 10:**
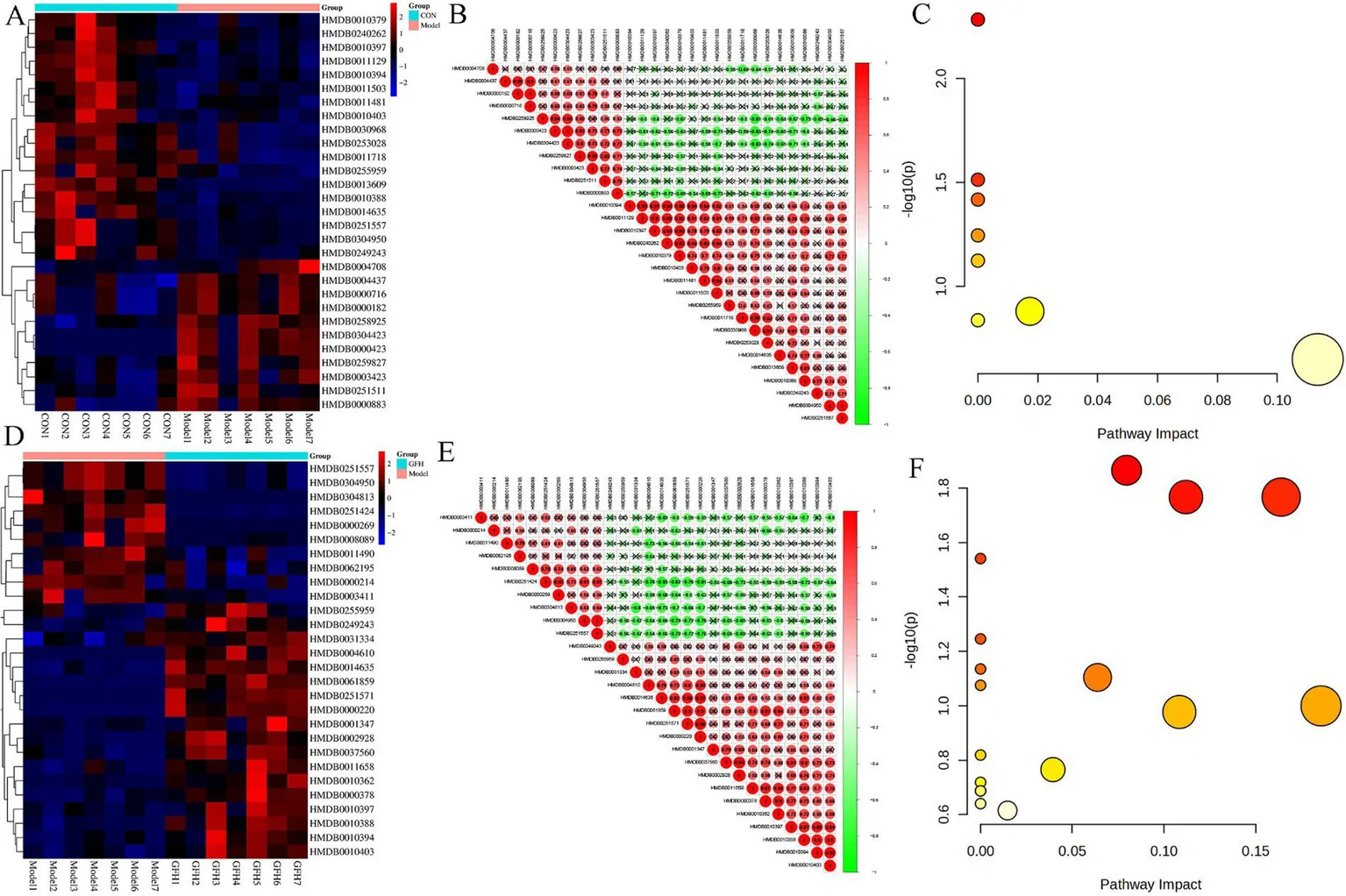
Heatmap of differential metabolite expression levels. **(A)** Differential metabolites between the Model and CON groups. **(B)** Correlation analysis of metabolites between the Model and CON groups. **(C)** Depression-induced metabolic pathway disorders in serum. **(D)** Differential metabolites between the GFH and Model groups. **(E)** Correlation analysis of metabolites between the GFH and Model groups. **(F)** Metabolic pathways involved in the antidepressant effects of GFH.

**Table 2:** Differential metabolites of Model vs. CON groups.

HMDB ID	HMDB name	Formula	Molecular mass	Retention time (min)
Vinylidene chloride	HMDB0259827	C2H2Cl2	95.95339	23.46
LysoPC(20:3(8Z,11Z,14Z)/0:0)	HMDB0010394	C28H52NO7P	545.3474	18.111
LysoPE(0:0/20:0)	HMDB0011481	C25H52NO7P	509.3476	19.081
LysoPE(16:0/0:0)	HMDB0011503	C21H44NO7P	453.2849	17.878
LysoPC(0:0/16:0)	HMDB0240262	C24H50NO7P	495.3318	17.943
LysoPE(0:0/18:0)	HMDB0011129	C23H48NO7P	481.3159	17.456
2,4,6-Octatriyn-1-ol	HMDB0030968	C8H6O	118.0419	1.972
3,4-Dihydroxyhydrocinnamic acid	HMDB0000423	C9H10O4	164.0473	1.739
3-Oxo-5beta-cholanoic acid	HMDB0304950	C24H38O3	374.2817	16.708
4-Hydroxybenzaldehyde	HMDB0011718	C7H6O2	122.0369	1.914
Bis(2-ethylhexyl) phthalate	HMDB0249243	C24H38O4	390.2766	13.794
Diethanolamine	HMDB0004437	C4H11NO2	105.0792	1.474
DL-Arginine	HMDB0251511	C6H14N4O2	174.1117	1.487
D-Glutamine	HMDB0003423	C5H10N2O3	146.0692	1.499
Lysine	HMDB0000182	C6H14N2O2	146.1056	1.309
D-Tryptophan	HMDB0013609	C11H12N2O2	204.0897	6.775
2,4,6,8,10,12-Docosahexaenoic acid, ethyl ester	HMDB0251557	C24H36O2	356.271	16.708
L-Pipecolic acid	HMDB0000716	C6H11NO2	129.079	1.299
LysoPC(14:0/0:0)	HMDB0010379	C22H46NO7P	467.3004	16.672
L-Valine	HMDB0000883	C5H11NO2	117.0791	1.624
LysoPC(18:3(9Z,12Z,15Z)/0:0)	HMDB0010388	C26H48NO7P	517.3162	16.908
LysoPC(20:5(5Z,8Z,11Z,14Z,17Z)/0:0)	HMDB0010397	C28H48NO7P	541.3157	16.567
LysoPC(22:5(7Z,10Z,13Z,16Z,19Z)/0:0)	HMDB0010403	C30H52NO7P	569.3466	17.834
N-benzylformamide	HMDB0304423	C8H9NO	94.04183	1.739
L-prolyl-L-leucine	HMDB0253028	C11H20N2O3	228.1474	1.826
9,12,13-TriHOME	HMDB0004708	C18H34O5	330.2425	20.29
Fosinopril	HMDB0014635	C30H46NO7P	563.2998	16.844
Olprinone	HMDB0255959	C14H10N4O	250.0849	8.624
tetranor-PGFM	HMDB0258925	C16H26O7	312.1634	16.908

**Table 3:** Differential metabolites of GFH vs. Model groups.

HMDB name	HMDB ID	Formula	Molecular mass	Retention time (min)
Glycerol alpha-monochlorohydrin	HMDB0031334	C3H7ClO2	110.0133	20.93
LysoPC(20:3(8Z,11Z,14Z)/0:0)	HMDB0010394	C28H52NO7P	545.3474	18.111
3,7,8,15-Scirpenetetrol	HMDB0037560	C15H22O6	298.1414	10.211
LysoPE(0:0/22:0)	HMDB0011490	C27H56NO7P	537.3798	22.135
PC(18:1(11Z)/22:5(7Z,10Z,13Z,16Z,19Z))	HMDB0008089	C48H84NO8P	833.5928	19.896
Phytosphingosine	HMDB0004610	C18H39NO3	317.2927	14.65
2-Methylbutyroylcarnitine	HMDB0000378	C12H23NO4	245.1626	8.029
3-Oxo-5beta-cholanoic acid	HMDB0304950	C24H38O3	374.2817	14.942
6-Hydroxy-5-methoxyindole glucuronide	HMDB0010362	C15H17NO8	339.0952	8.029
2,8-Dihydroxyquinoline-beta-D-glucuronide	HMDB0011658	C15H15NO8	337.0794	7.379
Bis(2-ethylhexyl) phthalate	HMDB0249243	C24H38O4	390.2766	13.794
Dinonyl phthalate	HMDB0251424	C26H42O4	418.3086	21.36
D-Proline	HMDB0003411	C5H9NO2	115.0635	1.704
2,4,6,8,10,12-Docosahexaenoic acid, ethyl ester	HMDB0251557	C24H36O2	356.2711	14.942
Dodecylamine	HMDB0251571	C12H27N	185.2143	15.271
Ornithine	HMDB0000214	C5H12N2O2	132.09	1.32
LysoPC(18:3(9Z,12Z,15Z)/0:0)	HMDB0010388	C26H48NO7P	517.3162	16.908
LysoPC(20:5(5Z,8Z,11Z,14Z,17Z)/0:0)	HMDB0010397	C28H48NO7P	541.3157	16.567
LysoPC(22:5(7Z,10Z,13Z,16Z,19Z)/0:0)	HMDB0010403	C30H52NO7P	569.3466	17.834
Methyl hexadecanoic acid	HMDB0061859	C17H34O2	287.2823	18.765
Palmitic acid	HMDB0000220	C16H32O2	273.2662	14.147
Platelet-activating factor	HMDB0062195	C26H54NO7P	523.3631	20.066
Sphinganine	HMDB0000269	C18H39NO2	301.2978	17.247
Xestoaminol C	HMDB0304813	C14H31NO	229.2404	14.053
Fosinopril	HMDB0014635	C30H46NO7P	563.2998	16.844
Isopentenyl pyrophosphate	HMDB0001347	C5H12O7P2	246.0096	7.105
Maltitol	HMDB0002928	C12H24O11	344.133	10.159
Olprinone	HMDB0255959	C14H10N4O	250.0849	8.624

#### 3.4.3 Integrated analysis of network pharmacology, metabolomics, and gut microbiota

By integrating network pharmacology with metabolomic profiling, we constructed compound-reaction-enzyme-gene networks (Figure 11A) to investigate the mechanisms underlying the antidepressant action of GF. Among the enriched pathways, glycosphingolipid metabolism emerged as a key pathway, in which PTEN was identified as the key target and phytosphingosine and sphinganine as the principal metabolites. Molecular docking was subsequently performed between quercetin and PTEN, and the resulting conformation was reliable and stable (Figure 11B), with a binding energy of −7.2 kcal/mol and an RMSD of 1.439, indicating spontaneous binding, with hydrogen bonds and van der Waals forces as the major intermolecular interactions (Figure 11C). Furthermore, correlation analyses were performed between the top 50 genera (ranked by relative abundance at the genus level) and the differential metabolites identified across groups (Figures 11D,E). The results showed that the vast majority of genera exhibited weak or negligible correlations with only a limited number of metabolites; in contrast, only a few genera, notably Bifidobacterium and Turicibacter, displayed pronounced correlations with a broad range of metabolites.

**Figure 11:**
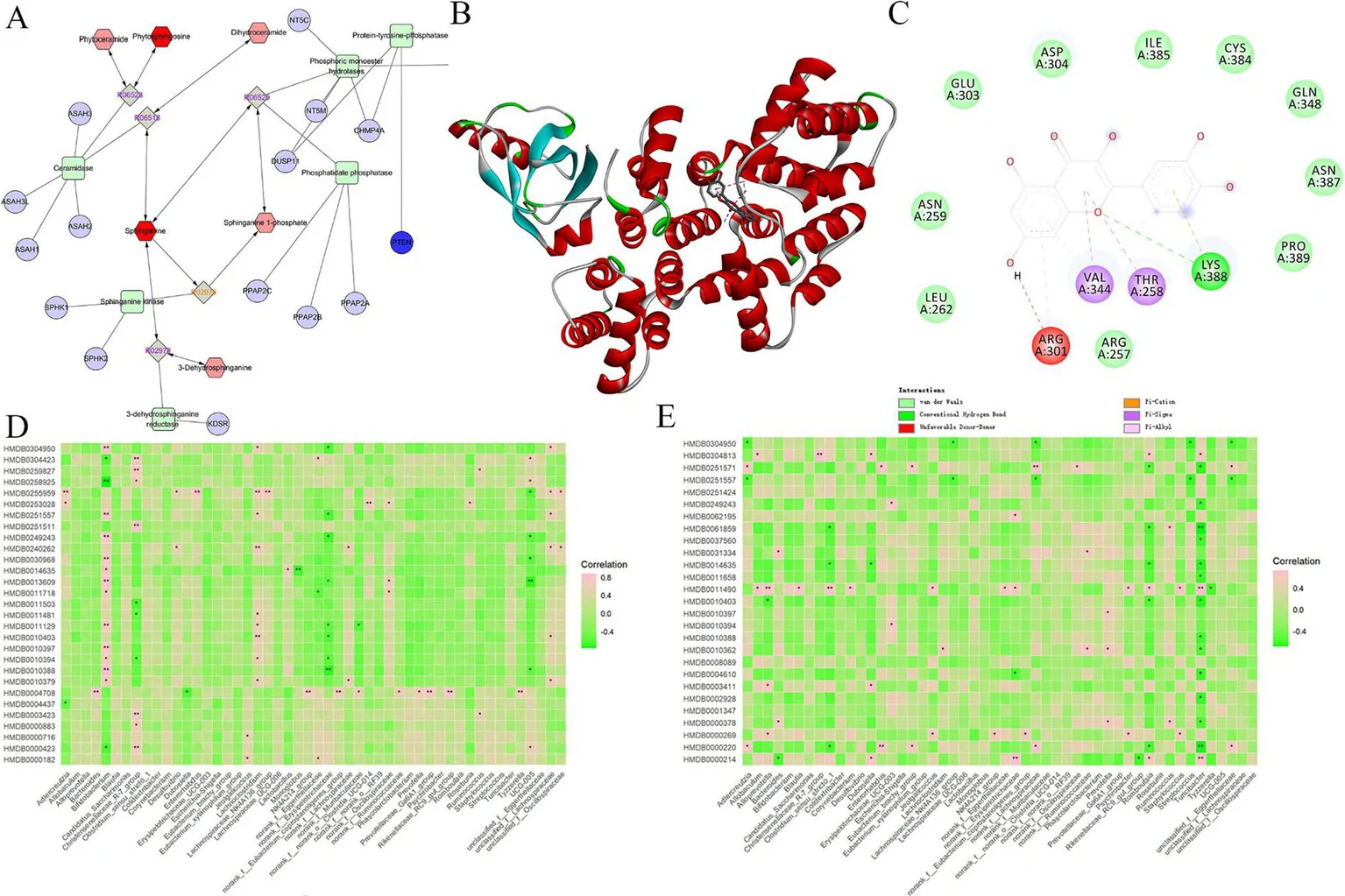
Integrated analysis of network pharmacology, gut microbiota, and metabolomics. **(A)** Glycosphingolipid metabolism. **(B)** Docking conformation of quercetin with PTEN. **(C)** Binding site. **(D)** Correlation analysis between gut microbiota and differential metabolites in the Model vs. CON groups. **(E)** Correlation analysis between gut microbiota and differential metabolites in the GFH vs. Model groups.

## 4 Discussion

Studies investigating disease mechanisms and treatment approaches through the lens of metabolomics and the gut microbiome have attracted growing interest (Ma et al., 2026; Dong et al., 2024). In the present study, we integrated gut microbiota, metabolomics, and network pharmacology to investigate possible mechanisms by which GF alleviates depressive-like behaviors. Our study revealed that GF not only markedly ameliorated depressive symptoms but also substantially modulated both serum metabolic profiles and gut microbial composition in depressive model rats. Previous studies have shown that GF can effectively ameliorate depression-like behaviors, and the present results were in agreement with those earlier reports (Tian et al., 2024; Liu et al., 2023; Ruan et al., 2019). Lu-Lu Zhang investigated the antidepressant efficacy and mechanism of GF at doses of 2, 4, and 6 g/kg (Zhang et al., 2026), and all doses exhibited pronounced antidepressant effects.

In this study, the differential metabolites in serum from depressed rats were primarily lipids, amino acids, and other metabolites. Lipids are major constituents of the brain. Among the tissues of the healthy adult human body, the brain is one of the richest in lipids. Lipids constitute roughly 12% of its fresh weight and as much as 50% of its dry mass. The structural and functional diversity of brain lipids is substantial. Principal lipid classes and subclasses occur in neurons as well as glial cells, a finding that reflects the different structural and functional demands of membrane organization, signaling pathways, and oxidative stress homeostasis. The human brain carries out a broad range of functions, extending from motor control to cognition, and all of these depend on the precise arrangement of distinct neuronal and glial populations. The presence of distinct lipid profiles in these cells contributes to their functional and morphological heterogeneity (Jové et al., 2023). We found numerous lipid metabolism disorders in serum from Model group rats, suggesting possible disturbances in cerebral lipid supply.

Among the major amino acids detected (DL-arginine, D-glutamine, lysine, D-tryptophan, and L-valine), L-valine is an indispensable amino acid and one of the branched-chain amino acids (BCAAs). Once taken up into the brain parenchyma, BCAAs perform various functions, including serving as fuel substrates for cerebral energy metabolism. In the central nervous system, they are essential for neurotransmitter synthesis, and perturbations in protein synthesis have been linked to neuronal disorders. Evidence accumulated to date suggests that an excess or deficiency of BCAAs may contribute to metabolic disturbances (Jové et al., 2023). The current investigation demonstrated that serum L-valine concentrations were significantly higher in Model group rats, suggesting impaired energy metabolism in these animals.

Integrated network pharmacology and metabolomics revealed that glycosphingolipid metabolism is the key pathway modulated by GF. As a crucial branch of lipid metabolism, this pathway has attracted extensive interest owing to its essential role in immune signaling. Glycosphingolipids are not only major structural components of cellular membranes but also act as dynamic signaling hubs involved in cellular stress responses, inflammatory responses, and various other biological processes.

Structurally, these complex lipids consist of a hydrophobic ceramide moiety and a hydrophilic oligosaccharide chain, the latter being assembled via the sequential addition of sugar residues to ceramide by linkage-specific glycosyltransferases. These molecules are widely distributed in eukaryotic plasma membranes and are involved in numerous essential biological processes, involving cell recognition, multiplication, specialization, growth control, and signal communication (Memon et al., 1999). Moreover, changes in the enzymatic activities of the glycosphingolipid metabolic pathway or abnormal metabolic fluxes of its intermediates have been shown to directly affect the *in vivo* expansion, cytotoxic capacity, and antitumor immunity of CD8^+^ T cells. Since their initial identification in brain tissue in 1942, glycosphingolipids have been recognized as important lipids that contribute to numerous physiological and pathological conditions. In particular, glycosphingolipid metabolism orchestrates the development and functional maturation of T cells and NK cells (Li et al., 2026). During neural development, the metabolic profile of glycosphingolipids undergoes reprogramming, shifting from globo-series to ganglio-series production. Disruption of this developmental switch has been linked to neurodevelopmental disorders in humans, highlighting the fundamental importance of glycosphingolipids in this context (Russo et al., 2018).

Glycosphingolipids act as immunomodulators and can initiate local and systemic inflammatory responses. They are widely distributed in the cells and biological fluids of vertebrates but are particularly highly enriched in nervous system. Their generation begins within endoplasmic reticulum compartment and is accomplished in Golgi apparatus. The abundance and expression patterns of glycosphingolipids undergo dramatic changes in the brain during embryonic development, later developmental stages, and stages after birth. Previous study has indicated that glycosphingolipids are predominantly enriched to plasma membrane microdomains and serve as critical constituents mediating signal pathways and cellular adhesion. During brain development, glycosphingolipids are also thought to contribute critically to nervous system formation. Disturbances in glycosphingolipid expression and metabolic profile can affect neurological function (Yu et al., 2009).

The target associated with glycosphingolipid metabolism is PTEN, a gene that serves an essential function in the brain. Individuals carrying PTEN mutations also exhibit numerous behavioral deficits involve impairments in motor function and fine motor skills, along with executive function, processing speed, working memory, and memory recall. Furthermore, people carrying PTEN mutations manifest pronounced sensory dysfunction, characterized by hyporesponsiveness and altered sound processing. From an anatomical perspective, PTEN mutation carriers show abnormal scaling in particular brain regions (Clipperton-Allen et al., 2022). In addition, PTEN is important for regulating neuronal activity. Evidence indicates that PTEN localized at the postsynaptic density binds to the synaptic scaffold protein PSD-95 via a PDZ-binding motif, and this association is essential for long-term depression mediated by N-methyl-D-aspartate receptors (Cheung et al., 2023). Furthermore, PTEN regulates multiple signal transduction events in the brain, including those required for central nervous system patterning, and is an important regulator of neuronal signaling. Mutations in PTEN alter neuronal morphology, soma size, and dendritic arborization (Ledderose et al., 2022). Quercetin, a major active component of GF, binds strongly to PTEN, suggesting that GF may exert a pronounced regulatory effect on neuronal activity. Sphinganine, an important metabolite in this pathway, belongs to the sphingolipid class. Sphingolipids are fundamental structural elements of eukaryotic cellular membranes and serve crucial functions in cell signaling and lipid raft organization. They participate in diverse cellular processes, including cell differentiation, pathogenesis, and apoptosis (Padilla-Gomez et al., 2022). In addition to their structural function, sphingolipids act as important bioactive mediators that modulate inflammation, immunity, autophagy, as well as cell growth and survival (Liu et al., 2026).

Correlation analysis between gut microbiota and metabolomics indicated that Bifidobacterium and Turicibacter exhibited significant correlations with multiple metabolites. Bifidobacterium has been demonstrated to exert potential neuroprotective effects (Li et al., 2024), in various experimental models, such as zebrafish, *Caenorhabditis elegans*, and even in clinical studies. *Bifidobacterium breve* can increase the levels of BDNF and intestinal short-chain fatty acids. Bifidobacterium can also increase plasma tryptophan levels and affect central serotonin transmission (Olajide and Ijomone, 2025), and specific Bifidobacterium strains can reduce abnormal inflammatory responses. It has been reported that Bifidobacterium strains possessing immunomodulatory activity which affect enteric and systemic inflammation, whereas decreased content of Bifidobacterium in human subjects or model systems were persistently related to aberrant immune responses. *Bifidobacterium longum* subsp. longum 1714™ has been investigated in a wide range of preclinical animal models and studies in humans. In studies of patients with irritable bowel syndrome, the combination of *B. longum* 1714 and *B. longum* 35,624™ reduced anxiety- and depression-related behaviors, which was accompanied by a reduction in TNF-α levels, a pro-inflammatory cytokine (Groeger et al., 2025). Turicibacter is an anaerobic Gram-positive bacterium belonging to the order Bifidobacteriales. As a principal abundant bacteria within gut microbiota, Turicibacter has been repeatedly reported to be pathogenic and is also associated with various pathological conditions in the body (Burakova et al., 2025). Turicibacter is closely related to inflammatory responses, such as those involving IL-6, ALT, and TNF-α (Liao et al., 2025). Inflammation is involved in the pathophysiology of depression, and enhanced inflammatory profiles can be observed in patients who respond poorly to conventional treatment. Inflammatory factors such as TNF-α are not only promising therapeutic targets but also valuable biomarkers for treating depression (Wu et al., 2026).

The current investigation is likewise subject to certain limitations. First, the network pharmacology coupled with molecular docking investigations were based solely on computational predictions. The active components, core targets, PPI network, GO/KEGG enrichment results, and quercetin-PTEN docking conformation were all derived from public databases and computational algorithms without experimental validation. Future studies should validate key targets using qPCR, western blotting, immunohistochemistry, and should confirm direct drug-target binding by cellular thermal shift assay surface plasmon resonance or isothermal titration calorimetry. Second, the active components of GF were identified solely through database mining, without experimental detection of compounds that actually enter the bloodstream; thus, the material basis of GF remains experimentally unverified. Some predicted components may be poorly absorbed, extensively metabolized, or unable to reach effective concentrations, whereas other unlisted compounds or metabolites may be the true bioactive effectors. Future work should use serum pharmacochemistry to identify serum prototype compounds and metabolites and to correlate their exposure with target engagement and pharmacodynamic effects.

## Data Availability

The data presented in the study are deposited in the NCBI repository (https://www.ncbi.nlm.nih.gov/), accession number: PRJNA1542039.

## Footnotes

1
https://www.genecards.org/

2
https://www.bioinformatics.com.cn/

3
https://cn.string-db.org/

4
http://www.hmdb.ca

5
http://www.lipidmaps.org

6
http://metlin.scripps.edu

7
http://www.genome.jp/kegg

8
https://www.rcsb.org/
